# On the Injurious Consequences of Unnecessary and Immoderate Bloodletting

**Published:** 1838-10

**Authors:** 


Art. VI.—*Die Nachtheile unzeitiger und uebermässiger Anwendung des Aderlasses und anderer Blutentziehung.* Von Dr. L. Wetzlar.—*Aachen*, 1837.

*On the Injurious Consequences of unnecessary and immoderate Bloodletting.* By Dr. Wetzlar.—*Aix la Chapelle*, 1837. 12mo. pp. 194.

Dr. Wetzlar seems to us to labour under an exaggerated apprehension of the ill effects of bloodletting, and that too even in cases and quantities in which its fitness is in this country considered as fully demonstrated. He has satisfied himself that there is still room for a new work on the above subject, notwithstanding the recent appearance of similar ones by his countrymen Schneider and Simon—works too which by no means minced the matter,—the one coming out under the redoubtable title of "*Hæmatomania*," and the other under the no less appalling one of "The *Vampirism* of the Nineteenth Century." Dr. W. commences his work with a comprehensive historical review of the variations of popularity to which this first of all remedies has been subjected. The new Italian school, and the active treatment of England, from Sydenham's time to our own days, are his peculiar aversion. The much talked of but little understood case of the lamented Malibran is adduced by him as triumphant evidence of the justice of his attack on English practice; a case, by the way, as to which he seems to be in possession of no accurate or authentic information, but to have rested satisfied merely with the newspaper accounts of the day; a source as to the value of which our English readers do not require to be enlightened. The school of Broussais, as it may be supposed, does not escape unscathed. Some of his anecdotes in regard to the excessive employment of leeches are sufficiently piquant; as, for example, that of a friend of the author, who being seized with the Parisian dysentery, so common to new-comers, was ordered by the

Coryphæus of the sect to put eighty leeches to the abdomen; an application at which his German prudence having revolted, pastilles of ipecacuanha along with simple mucilaginous drinks were substituted with the happy effect of speedily removing the disorder! The editor of the Drapeau blanc, less judicious or less fortunate, applied no less than five hundred leeches to his gouty finger in successive days, without having any diminution of his sufferings to congratulate himself on, even after the last of them had dropped off.

Dr. W. has some good remarks on epidemic and endemic constitutions in relation to the effects of bloodletting. In respect to the latter, he contrasts the town of Aix la Chapelle, where he is resident, with Bonn, which is at no very great distance from it, bloodletting being much worse borne at the former than at the latter, the situation of which is more open and airy.

He dwells at considerable length on the fallacious indications for venesection drawn from certain pains of the head, chest, and abdomen, and, we think, very judiciously. The following picture is not exaggerated.

"Stitch in the side may depend on mere congestion, and that, moreover, often of a merely passive kind; thus chlorotic patients are frequently affected with a violent pain of this kind combined not unusually with cough and difficulty of breathing. But the pulse is here generally a sufficient means of diagnosis, being seldom or ever hard, whilst at the same time the effort of speaking does not exasperate the pain, which is usually seated beneath the false ribs. In spite of the chlorotic condition, many physicians bleed in such cases; and a momentary improvement may be the result, but only so far as the mere pain in the side is concerned; the general strength is depressed still farther, the stitch speedily returns, and under the repeated employment of the supposed remedy, the patient soon sinks consumptive or dropsical."

